# Comparison between Three Radiomics Models and Clinical Nomograms for Prediction of Lymph Node Involvement in PCa Patients Combining Clinical and Radiomic Features

**DOI:** 10.3390/cancers16152731

**Published:** 2024-07-31

**Authors:** Domiziana Santucci, Raffaele Ragone, Elva Vergantino, Federica Vaccarino, Francesco Esperto, Francesco Prata, Roberto Mario Scarpa, Rocco Papalia, Bruno Beomonte Zobel, Francesco Rosario Grasso, Eliodoro Faiella

**Affiliations:** 1 Department of Diagnostic Imaging, Campus Bio-Medico University of Rome, 00128 Rome, Italy; raffaele.ragone@unicampus.it (R.R.); elva.vergantino@unicampus.it (E.V.); federica.vaccarino@unicampus.it (F.V.); b.zobel@policlinicocampus.it (B.B.Z.); r.grasso@policlinicocampus.it (F.R.G.); e.faiella@policlinicocampus.it (E.F.); 2 Department of Urology, Campus Bio-Medico University of Rome, 00128 Rome, Italy; f.esperto@policlinicocampus.it (F.E.); f.prata@policlinicocampus.it (F.P.); r.scarpa@policlinicocampus.it (R.M.S.); rocco.papalia@policlinicocampus.it (R.P.)

**Keywords:** artificial intelligence, ChatGPT, multiparametric magnetic resonance imaging (mp-MRI), prostate cancer (PCa), logistic regression (LR), random forest (RF), support vector machine (SVM), clinical nomogram

## Abstract

**Simple Summary:**

Our study aims to demonstrate and quantify the usefulness of three different AI models for lymph node involvement prediction in prostate cancer patients comparing the performance with current routinely used clinical nomograms. Knowing the lymph node involvement with accuracy and confidence can influence the type of surgical and therapeutic choice.

**Abstract:**

PURPOSE: We aim to compare the performance of three different radiomics models (logistic regression (LR), random forest (RF), and support vector machine (SVM)) and clinical nomograms (Briganti, MSKCC, Yale, and Roach) for predicting lymph node involvement (LNI) in prostate cancer (PCa) patients. MATERIALS AND METHODS: The retrospective study includes 95 patients who underwent mp-MRI and radical prostatectomy for PCa with pelvic lymphadenectomy. Imaging data (intensity in T2, DWI, ADC, and PIRADS), clinical data (age and pre-MRI PSA), histological data (Gleason score, TNM staging, histological type, capsule invasion, seminal vesicle invasion, and neurovascular bundle involvement), and clinical nomograms (Yale, Roach, MSKCC, and Briganti) were collected for each patient. Manual segmentation of the index lesions was performed for each patient using an open-source program (3D SLICER). Radiomic features were extracted for each segmentation using the Pyradiomics library for each sequence (T2, DWI, and ADC). The features were then selected and used to train and test three different radiomics models (LR, RF, and SVM) independently using ChatGPT software (v 4o). The coefficient value of each feature was calculated (significant value for coefficient ≥ ±0.5). The predictive performance of the radiomics models and clinical nomograms was assessed using accuracy and area under the curve (AUC) (significant value for *p* ≤ 0.05). Thus, the diagnostic accuracy between the radiomics and clinical models were compared. RESULTS: This study identified 343 features per patient (330 radiomics features and 13 clinical features). The most significant features were T2_nodulofirstordervariance and T2_nodulofirstorderkurtosis. The highest predictive performance was achieved by the RF model with DWI (accuracy 86%, AUC 0.89) and ADC (accuracy 89%, AUC 0.67). Clinical nomograms demonstrated satisfactory but lower predictive performance compared to the RF model in the DWI sequences. CONCLUSIONS: Among the prediction models developed using integrated data (radiomics and semantics), RF shows slightly higher diagnostic accuracy in terms of AUC compared to clinical nomograms in PCa lymph node involvement prediction.

## 1. Introduction

Prostate cancer ranks among the most prevalent malignancies tumors and stands as the second leading cause of cancer-related deaths in men [1]. In 2023, almost 2 million new cancer cases and more than 600,000 cancer deaths occurred in the United States [2,3].

The identification of pelvic lymph node metastasis (PLNM), present in approximately 15% of newly diagnosed prostate cancer (PCa) patients, stands as a crucial prognostic factor correlating with biochemical recurrence and distant metastases after curative treatment. Therefore, accurate identification of PLNM before treatment for localized PCa would significantly impact clinical decision-making, treatment planning, and the ability to predict outcomes for patients.

According to the European Association of Urology (EAU) guidelines, pelvic lymph node dissection (PLND) or extended pelvic lymph node dissection (ePLND) are considered the most precise staging procedure for pelvic lymph node metastasis (PLNM) assessment in PCa patients. Nonetheless, it is important to note that PLND carries an elevated risk of complications in 20% of patients, causing significant morbidity [4,5].

PLND or ePLND are advised for patients with intermediate and high-risk PCa when the estimated risk of positive lymph nodes exceeds 5% of Briganti nomograms [6,7].

Several nomograms have been introduced by EAU and the National Comprehensive Cancer Network (NCCN) to predict lymph node invasion (LNI) and aid clinicians in determining the necessity of PLND during radical prostatectomy. These models demonstrate satisfactory performance [7,8,9,10,11]. Achieving an accurate, noninvasive, and preoperative detection and characterization of lymph node status is crucial for clinicians when deciding whether to proceed with PLND [8]. The most widely used and validated scoring models used nowadays are Briganti [9], MSKCC [10], Partin [11,12,13,14], Yale, and Roach [10,11], which include the evaluation of the PSA, the Gleason score, TNM staging, and histological findings after biopsy.

Over the last few decades in the PCa imaging analysis field, several researches on the role of artificial intelligence (AI) have been conducted [15,16,17,18,19]. Among AI approaches, the radiomics ones have been more deeply investigated, with the aim to predict nodule and tumor behavior without invasive procedures. This approach has the potential to overcome certain limitations in diagnostic accuracy associated with human interpretation. Recently, radiomics analysis has gained prominence for offering a more quantitative and objective assessment of medical images. Radiomics features, extracted from the MRI tumor, may capture histopathological characteristics, providing prognostic information for cancer management through the analysis of quantitative aspects of tumor intensity and shape.

The purpose of this study is to evaluate the performance of three different proposed radiomics models in the lymph node invasion prediction in PCa patients, comparing the results with current clinical nomograms.

## 2. Materials and Methods

This study was conducted according to the guidelines of the Declaration of Helsinki. An ethical review and approval were waived for this study, due to its retrospective nature.

All patients who received mp-MRI for staging based on prostatic tumor suspicion, from 2016 to 2022, at our Radiological Department, were retrospectively reviewed. Only patients who received prostatectomy and lymphadenectomy were included, for a total sample of 95 patients. 

All patients with PCa who received hormonal and/or radiation therapy or random biopsy before mp-MRI execution were excluded.

The prostatectomy and lymphadenectomy data were used for histological analysis, which is used as gold standard in our work. Patient clinical data (age, PSA before mp-MRI), tumor MRI characteristics (signal intensity in T2 weighted images; signal intensity in DWI/ADC map; PIRADS score), and histological tumor details (Gleason Score (GS); TNM staging, including invasion of the capsule, invasion of the seminal vesicles, and involvement of the neuro-vascular bundle; and histological type) were collected for each patient. All these parameters were defined as “semantic features”. The sample was divided into the following two groups based on lymph node status: positive, if at least one lymph node was involved at lymphadenectomy analysis (*n* = 30), and negative, if all examined lymph nodes were safe from metastases (*n* = 65).

Basing on clinical–pathological data, the following clinical nomograms were calculated using referred websites: Briganti [9], MSKCC [10], Partin [11,12,13,14], Yale, and Roach [10,11].

Details are discussed in the followed paragraphs.

### 2.1. Magnetic Resonance Imaging

A Siemens 1.5 T magnet was used. The mp-MRI protocol consisted of multiplanar T2-weighted images, an echo-planar DWI with b-values of 0, 800, or 1000 s/mm^2^ (ADC maps were automatically calculated), dynamic contrast-enhanced imaging (DCE), and Axial T1-weighted images.

During the exam, the patient was positioned supine and an external coil was employed, with a field that included the prostate gland, seminal vesicles, and pelvis up to the aortic bifurcation. For all patients, preoperative prostate mp-MRI scanning was performed 4–5 weeks after ultrasound-guided transrectal biopsy.

All prostate MRI examinations were analyzed in agreement by two radiologists with 12 years (E.F.) and 5 years of experience (D.S.) and the target lesion was identified in all the sequences, measured, and captured as a key image.

### 2.2. Segmentation, Feature Extraction

**Figure 1:**
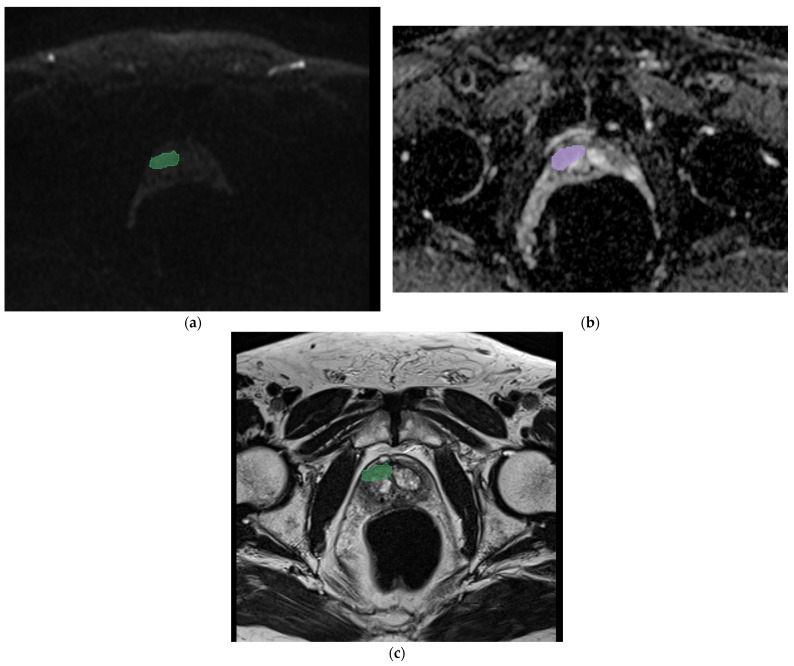
Segmentation of the index prostatic nodule (green zone) in DWI (**a**), (violet zone) ADC map (**b**), and (green zone) axial T2 (**c**), in a 54-year-old patient with a PSA value of 6.1 ng/mL. The histological analysis showed an adenocarcinoma with a Gleason score of 8 (4 + 4), with a positive margin of resection and four lymph nodes at lymphadenectomy. In this case, the random forest applied on DWI sequences predicts the involvement of lymph nodes with a confidence of 86%, a logistic regression of 67%, and an SVM of 30%.

All mp-MRI exams were downloaded and transferred onto a dedicated workstation. Then, a manual segmentation of the prostate nodule was performed by a resident with 3 years of experience (R.R.) using an open-source segmentation program (3D Slicer v. 5.0.1 [20]) on T2w sequences, DWI, and ADC maps. Then, a volume of interest (VOI) was obtained for each mask (see Figure 1).

For each VOI, first-order and second-order features were thus extracted using the Pyradiomics application v. 3 of the same software and automatically selected.

For all the analysis, both semantic features and radiomics features (Absolute Gradient shape, first order, Gray-Level Cooccurrence Matrix (GLCM), Gray Level Dependence Matrix (GLDM), Gray-Level Run-length Matrix (GLRLM), Gray-Level Size Zone Matrix (GLSZM) and Gray-Level Distance Zone Matrix (GLDZM)) were included. In particular:

- Shape features: These describe the geometric properties of the region of interest (ROI), such as the surface area, total volume, diameter, elongation, sphericity, and surface-to-volume ratio.
- First-order statistics (histogram-based features): These detail the distribution of voxel intensities within the image ROI, using conventional parameters like energy, entropy, mean, interquartile range, skewness, kurtosis, and uniformity.
- Second-order statistics (textural features): These capture the statistical interrelationships between neighboring voxels. Notable methods include:
  ○ Gray-level Cooccurrence Matrix (GLCM): Analyzes the spatial distribution of gray-level intensities in a 3D image.
  ○ Gray-Level Run-length Matrix (GLRLM): Measures contiguous voxels with the same gray-level value, characterizing the gray-level run lengths in various directions.
  ○ Gray-Level Size-Zone Matrix (GLSZM): Quantifies the zones of connected voxels sharing the same gray-level intensity in a 3D image.
  ○ Neighboring Gray-Tone Difference Matrix (NGTMD): Calculates the difference between a voxel’s gray value and the average gray value of its neighbors within a specified distance.
  ○ Gray-level Dependence Matrix (GLDM): Assesses the number of connected voxels within a certain distance that depend on the center voxel.

A coefficient value was calculated for all selected features considering the T2 sequences for all the three different models and considering the ADC and DWI sequences for only the RF and LR models (SVM was not possible to evaluate due to data complexity). The results were considered significant for a coefficient value higher than ±0.5.

### 2.3. Radiomics Analysis and Model Development

The development of the artificial intelligence models was implemented in the following three phases: selection of features, models development with features extraction, and models performance tests.

ChatGPT artificial intelligence was used for data analysis. Logistic regression (LR), random forest (RF), and support vector machine (SVM) were employed as radiomics models.

The performance of the model was evaluated in terms of accuracy and area under the curve ROC (AUC).

### 2.4. Clinical Nomograms

The Briganti, MSKCC, Yale, and Roach nomograms were calculated considering the data for each individual patient and applying the relevant formulas, available open source online.

In detail, the following criteria were used:

- Briganti: Preoperative PSA, clinical stage T, Gleason score of the biopsy, percentage of positive cores with a high level of prostate cancer, percentage of positive cores with a low level of prostate cancer.
- MSKCC: Age, thickness, Clark level, localization, ulceration.
- Yale: PSA, Gleason score, clinical T stage.
- Roach: PSA, Gleason score.

A comparison between the radiomics models’ performance and the nomograms’ performance was conducted using Bootstrapping and Harley McNeil tests, with the significant value set at *p* ≤ 0.05. The single sequence was compared with the clinical nomograms both one vs. one and all vs. all.

## 3. Results

All patients’ characteristics are showed in Table 1.

For each patient, three volumes of interest (VOIs) were obtained by the segmentation of the tumor lesion on the T2 sequence, DW images, and ADC maps.

From each VOI, 113 radiomics features were finally selected for each individual mask (including 18 first-order statistical features, 14 shape-based features, and 81 texture features using the PyRadiomics package in Python 3).

In total, a total of 343 features (14 semantic and 339 radiomics) were considered per patient and processed using ChatGPT.

The five most significant features and the five less significant features for the applied models in predicting lymph node involvement are shown in Table 2a–c for T2, DWI, and ADC, respectively.

In particular, the most significant radiomics features obtained were T2_nodulofistorderVariance (median 6.881.386.633.418.290), with a positive coefficient value equal to 0.4459, and T2_nodulofirstorderKurtosis (median 5.612.998.068.796.730), with a negative coefficient value equal to −0.5975, both obtained through the logistic regression model analysis.

Table 3 shows the performance of each model analyzed. In particular, the models that have greater overall predictive power are DWI in random forest (86% accuracy and 0.89 AUC) and support vector machine (89% accuracy and 0.28 AUC), and ADC in random forest (89% accuracy and 0.67 AUC).

The performance of the clinical nomograms was reported in Table 3. 

The two statistical tests, Bootstrapping and Harley McNeil, demonstrated the higher performance of the RF model for DWI analysis compared to all nomograms (with a *p* value ≤ 0.05 for YALE and MSKCC and close to 0.05 for Briganti) and the superiority of all nomograms compared to SVM and LR, in predicting lymph node invasion.

No other statistical significance in AUC were observed when radiomics-proposed models and nomograms were compared (Table 4a–c).

**Figure 2:**
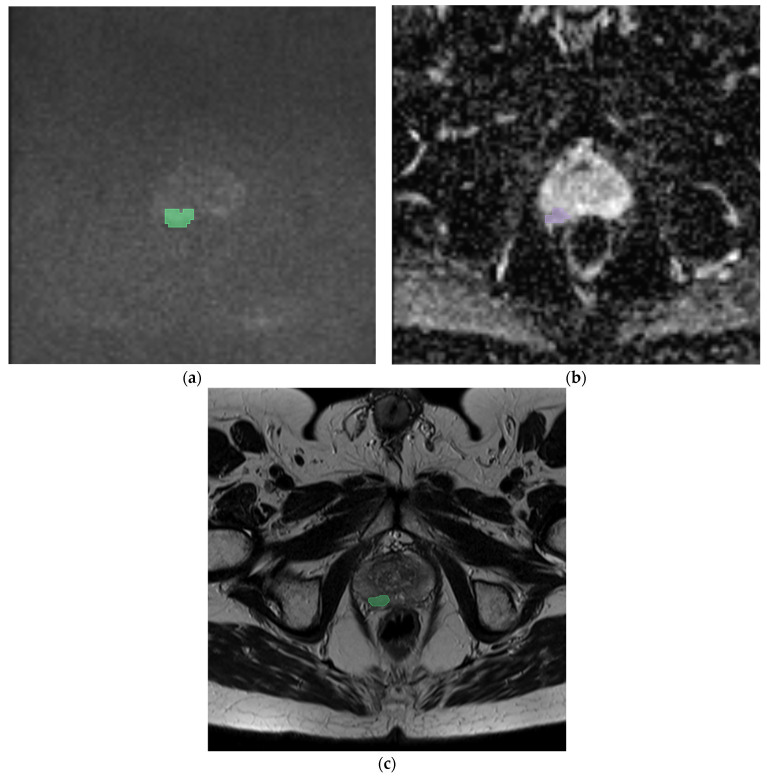
Prostatic nodule (green zone) in DWI (**a**), (violet zone) ADC map (**b**), and (green zone) axial T2 (**c**), in a 55-year-old patient with a PSA value of 2.5 ng/mL. The histological analysis showed an adenocarcinoma with a Gleason score of 6 (3 + 3), without lymph node involvement at lymphadenectomy. In this case, the random forest in DWI predicts the involvement of lymph nodes with a confidence of 85%, a logistic regression of 69%, and an SVM of 32%.

We reported two exemplary cases in Figure 1 and Figure 2. In the first case, we analyzed the features extracted from a prostatic tumor nodule of a 54-year-old patient with a PSA of 6.1 ng/mL with an adenocarcinoma Gleason score of 8 (4 + 4), a positive margin of resection, and four positive lymph nodes. In the second case, we analyzed the features of a 55-year-old patient with a PSA of 2.5 ng/mL with an adenocarcinoma Gleason score of 6 (3 + 3), without lymph node involvement. In both cases, the analysis performed showed the maximum accuracy using the RF model applied on the DWI sequences (86% and 85% of AUC, respectively). The features that showed the coefficient with best negative and positive prediction were T2_nodulofirstorderKurtosis and T2_nodulofirstorderVariance, respectively. These two features were the same features that also demonstrated the highest coefficient in the generic analysis, as shown in Table 2a.

## 4. Discussion

In this study, we show the capability of three different AI models to identify positive or negative lymph node involvement in patients with a PCa diagnosis, using mp-MRI features extracted from a prostate cancer nodule. We demonstrate that radiomic features have a slightly higher accuracy in terms of AUC compared to clinical nomograms.

The current clinical guidelines recommend lymphadenectomy as the primary method for lymph node involvement detection [16,17,18,19,20,21]. Specifically, the European Association of Urology (EAU), the European Society for Radiotherapy and Oncology (ESTRO), the EAU Section of Urological Research (ESUR), and the International Society of Geriatric Oncology (SIOG) suggest an extended pelvic lymph node dissection (e-PLND) for patients with over a 5% risk of nodal involvement. Similarly, the American Urological Association (AUA), American Society for Radiation Oncology [22] (ASTRO), and Society of Urologic Oncology (SUO) advocate for PLND in all intermediate- to high-risk patients, with PLND consideration also for localized PCa. The National Comprehensive Cancer Network (NCCN) guidelines recommend e-PLND for patients with more than a 2% risk of nodal metastasis [15,16].

According to these guidelines, lymphadenectomy should be conducted in the same session of radical prostatectomy for advanced-stage PCa at diagnosis. The procedure is typically performed at specific lymph node sites, including the internal and external obturator and inguinal nodes. A major issue, however, is the complications that often follow lymphadenectomy. To reduce the complications rate and limit the non-necessary lymphadenectomy, different algorithms that use clinical and anatomopathological data are used to estimate the likelihood of lymph node involvement and patient prognosis. The most employed clinical nomograms are Briganti, Yale, Roach, and MSKCC. Nowadays, their role is well established. In particular:

- The Briganti model [23] is used for cancer involvement and grading heterogeneity in biopsy samples and improves the accuracy in estimating the risk of lymph node invasion (LNI), suggesting changes in staging approaches.
- Due to the risk of overtreatment with the only Briganti model, the MSKCC (Memorial Sloan Kattering Cancer Center) calculate another nomogram with a minimalist approach. In this case, the evaluation includes only the PSA, age, and biopsy Gleason score.
- The Yale is a linear model based on PSA levels, the T stage, and the Gleason score. This model more successfully classifies high-risk categories patients (>15%). Unlike previous models, Yale does not underestimate the risks associated with lymph node involvement.
- The Roach formula [24] that includes only the PSA and the Gleason score can be used for the evaluation of lymph node involvement and the seminal vesicle and capsular involvement.

Regarding the imaging, multiparametric MRI (mp-MRI) is the most accurate technique for PCa detection, characterization, and staging definition and it is also performed for early-stage tumor evaluation, even when the prostate nodule is not detectable on transrectal ultrasound [17,25,26] (TRUS) or through palpation. The high sensitivity of T2 sequences for the transition zone (94%) and of DWI sequences for the peripheral zone (96%), followed by biopsy, facilitate this process [27,28,29]. This early assessment enables a more streamlined and precise planning of the therapeutic approach [16], tailored to the patient.

Nevertheless, imaging’s role in detecting and characterizing lymph nodes in cancer remains limited. While MRI sequences can identify lymph nodes larger than 5 mm, the technique often fails to detect micrometastases. Surgical experience then dictates the approach as follows: either a preventive lymphadenectomy with subsequent pathological staging or no intervention. These decisions are still primarily based on the clinical guidelines, which have yet to be extensively substantiated in the literature [18,19,20,21,22,23,24,25,27,28,29,30].

For these reasons, recent literature [28,29] has focused on the preoperative evaluation of lymph nodes affected by cancer other methods, which could be obtained using quantitative data. Advances in artificial intelligence and radiomics have significantly improved data quality, enabling a more refined assessment of these elements.

Radiomics features, including shape, contours, and various first- and second-order features related to the intensity of grayscale and other aspects, are increasingly used in the evaluation of various cancers, including PCa [30,31,32], offering a nuanced view of tumor characteristics, aiding in an accurate assessment, and aiding in understanding cancer progression and treatment planning [33].

In this work, we aim to evaluate the application of three machine learning models for the lymph node status prediction in PCa patients to assess the necessity of lymphadenectomy during surgery [34].

The features were extracted by the main lesions using the same mask for the three MRI sequences (T2, ADC, DWI). Among these, the features with the highest importance index were selected, meaning only those with the greatest impact on predicting lymph node involvement, and used to build and train the models.

In predictive terms, some features have a high positive predictive value, correlating the high absolute value of individual data with a high probability of lymph node involvement. Conversely, other features have a high negative predictive value, inversely correlating with a low probability of lymph node involvement. Additionally, the closer the absolute value of a feature is to one, the more significant its weight in the direct or inverse probability of lymph node involvement; the closer to zero, the less significant its weight. The most significant features identified in our study were the T2_nodulofirstorderKurtosis (−0.5975) and T2_nodulofirstorderVariance (+0.4459). Both features belong to the first-order class and were identified for T2 sequences. This could be explained because the T2 are the sequences with the highest contrast resolution and the first-order features are descriptors of the intensity distribution of the voxels with basic and widespread metrics. As expressed in literatures, the T2 sequences [33,34] can predict the PCa aggressiveness and biochemical recurrence. In particularly, Nketiah et al. [35] demonstrated that among the T2w extracted features, the textures ones could be used as diagnostic markers to pathological sequences, with the highest power for the combination with the Gleason score.

The single class of features selected for analysis conducted on ADC and DWI sequences do not reach significant coefficient values.

For the analysis of the extracted features, we employed different traditional radiomics models for each of the masks considered (then, for all the three sequences examined T2, DWI, and ADC) and we compared their performance with the most used clinical nomograms for predicting lymph node status in PCa patients.

Random forest, logistic regression, and support vector machine models were selected for radiomics analysis. Random forest [35,36] builds on decision trees, employing an ensemble method to overcome their limitations like overfitting and sensitivity to data variance by averaging numerous tree predictions, each trained on random data and feature subsets. This improves robustness and handles complex feature interactions, suitable for various classification and regression tasks. Logistic regression [37,38,39,40] offers a linear approach to model the dependency between features and the outcome, ideal for binary classification. It uses a weighted sum of features, applying a sigmoid function to provide a probabilistic output, which is straightforward but may struggle with more complex data relationships. A support vector machine [9,11,41,42] (SVM) typically constructs a hyperplane in a high-dimensional space to separate different classes, optimizing the margin between them. An SVM is effective for both linear and nonlinear classification, relying on kernel functions to manage complex data sets.

For each model, the area under the curve (AUC) and accuracy values were calculated for predicting positive lymph nodes.

When comparing the best performance of radiomics models to clinical nomograms, a statistically significant higher AUC was demonstrated only for DWI in RF, compared to the Briganti, MSKCC, and Yale nomograms (0.89 vs. 0.79 and 0.78, respectively) with a statistical difference. On the other hand, while for ADC there was no significant difference in performance between radiomics and clinical nomograms, the evaluation on T2 showed the superiority of clinical nomograms when compared to SVM.

Liu et al. [43] examined 128 individuals with PCa and pelvic lymph node metastasis (PLNM) and compared four radiomics models only in ADC masks (AUC 0.73, 0.63, 0.70, 0.56) with the MSKCC and Briganti 2017 nomograms and PIRADS (AUCs of 0.71, 0.70, and 0.70, respectively) with no statistically significant differences. Their results are in accordance to our study.

In another study, Liu et al. [44] built two preoperative PLNM prediction models using multivariate logistic regression and including radiological and radiomics characteristics, obtaining an AUC of 0.89–0.90, similar to our best study results. Moreover, they obtained that, for the purpose of PLNM prediction in PCa patients, a DWI-based radiomics nomogram that combines the LN radiomics signature with quantitative radiological features appears promising, especially for normal-sized LNM; in our study, our models based on DWI features obtained, coherently with Liu et al. [41], an accuracy of 0.67 and 0.89, respectively, for the LR and RF models.

Concerning the best AUCs, our radiomics models showed a performance of 0.89 for RF analysis using DWI sequences and for LR using T2 sequences and 0.78 for RF analysis using T2 sequences. Zheng et al. [45] used an integrated radiomics model (IRM) made of the histopathologic investigation, integrating radiomics features retrieved from a prostatic index lesion, and clinical features using only an SVM to confirm the predictive power for LNI. The testing set’s AUC for the suggested IRM was 0.915; in addition, IRM AUC was higher than the relative clinical nomograms (0.698–0.724) with a statistically significant difference (*p* ≤ 0.05). These results are extremely high compared to our results, considering the prevalence in performance of the clinical nomograms for our SVM machine analysis for T2 sequences. This may be due to a larger data set (244 PCa), but a smaller number of features for each patient (220); moreover, they used five cross-validations and a 3T MRI and included only ADC and T2 sequences.

Our study had several limitations. First, the small number of patients for the initial data set. Second, the complexity of data that cannot be evaluated, particularly with the SVM model with ChatGPT.

## 5. Conclusions

Despite the promising results, we are not yet able to replace clinical nomograms with prediction through artificial intelligence of lymph node status in prostate cancer patients.

However, the works present in the literature so far are rather discordant, both in the use of radiomics models and in the type of sequence. In the future, our research group will focus on evaluating individual magnetic resonance imaging sequences (also considering the T2 of the entire prostate and the T2 of the periprostatic fat) and the differences between the various models used (RF, LR, and SVM) in assessing lymph node involvement in prostate cancer patients.

In relation to the growing interest in noninvasive and increasingly personalized and conservative medicine, AI offers an extremely interesting and flexible panorama that can offer interesting solutions. This justifies the growing research and collective commitment, which increasingly requires data sharing and comparison.

## Figures and Tables

**Table 1:** Patients’ characteristics.

Category	Number
Number of patients	95
Patients with metastatic nodule at lymphadenectomy	35
Patients without metastatic nodule at lymphadenectomy	60
Race	Caucasian
Age	40–80
PSA [ng/mL] (Median, range)	4.5 (7.1)
Period of mp-MRI	2016–2023
Gleason Grade (Mediana)	7
Tumor target Zone Peripheral	33
Tumor target Zone Transition	12
Disease Grade	T3 (16) T2 (10) T3,5 (19)

**Table 2:** (**a**–**c**) For each patient, 113 radiomics features were extracted. In this table, the five features with the most positive (1–5) and most negative predictive coefficient (6–10) were reported. (**a**) The top five most influential features of radiomics analysis, using only the T2 nodule as the parameter. The relative coefficient for the three models were reported for each feature (LRcoefficient for LR analysis, SVMcoefficient for SVM analysis, and RFImportance for RF analysis). The most significant ones are the ones that are closer to 1. (**b**) The top five most influential features of radiomics analysis, using only the DWI nodule as the parameter. The relative coefficient for the two adopted models were reported for each feature (LRcoefficient for LR analysis and RFImportance for RF analysis). The most significant ones are the ones that are closer to 1. (**c**) The top five most influential features of radiomics analysis, using only the DWI nodule as the parameter. The relative coefficient for the two adopted models were reported for each feature (LRcoefficient for LR analysis and RFImportance for RF analysis). The most significant ones are the ones that are closer to 1.

**(a)**				
**#**	**Features**	**LogRegCoef**	**SVMCoef**	**RFImportance**
1	T2_noduloglcmContrast	0.0084	0.0427	0.0457
2	T2_nodulofirstorderKurtosis	−0.5975	−0.2378	0.0395
3	T2_nodulofirstorderMeanAbsoluteDeviation	−0.0009	0.0231	0.0364
4	T2_nodulofirstorderVariance	0.4459	0.1540	0.0332
5	T2_noduloglcmIdm	0.1337	0.0445	0.0320
6	T2_noduloshapeSphericity	0.0420	0.0148	0.0000
7	T2_nodulofirstorder10Percentile	−0.1215	−0.0385	0.0000
8	T2_noduloglcmDifferenceVariance	−0.3277	−0.1183	0.0000
9	T2_noduloglrlmGrayLevelNonUniformityNormalized	0.0420	0.0148	0.0000
10	T2_noduloglrlmLowGrayLevelRunEmphasis	0.0420	0.0148	0.0000
**(b)**				
**#**	**Feature**	**RFImportance**		**LogRegCoef**
1	DWIfirstorderEnergy	0.0770		3.50 × 10^−8^
2	DWIglcmIdn	0.0693		−4.46 × 10^−13^
3	DWIglrlmRunLengthNonUniformityNormalized	0.0663		−1.39 × 10^−13^
4	DWIglszmGrayLevelNonUniformityNormalized	0.0592		−2.85 × 10^−13^
5	DWIgldmSmallDependenceEmphasis	0.0507		9.34 × 10^−15^
6	DWIfirstorderMedian	0.0481		−2.17 × 10^−11^
7	DWIglrlmGrayLevelNonUniformityNormalized	0.0445		−2.95 × 10^−13^
8	DWIglcmImc1	0.0429		5.94 × 10^−14^
9	DWIglcmIdmn	0.0423		−3.91 × 10^−13^
10	DWIglcmCorrelation	0.0410		−1.85 × 10^−13^
**(c)**				
**#**	**Feature**	**RF Importance**		**LogRegCoef**
1	ADCglszmGrayLevelNonUniformityNormalized	0.0339		1.25 × 10^−33^
2	ADCglcmSumAverage	0.0320		−3.62 × 10^−15^
3	ADCshapeMeshVolume	0.0297		−1.07 × 10^−15^
4	ADCfirstorderUniformity	0.0274		−7.88 × 10^−33^
5	ADCshapeMajorAisLength	0.0251		3.17 × 10^−15^
6	ADCfirstorderMeanAbsoluteDeviation	0.0249		1.27 × 10^−15^
7	ADCshapeMaimum2DDiameterSlice	0.0231		2.15 × 10^−15^
8	ADCglszmSmallAreaLowGrayLevelEmphasis	0.0228		1.56 × 10^−32^
9	ADCglrlmRunVariance	0.0222		9.95 × 10^−32^
10	ADCglcmImc1	0.0222		7.52 × 10^−32^

**Table 3:** Performance of the four radiomics models. The accuracy and AUC of the various prediction models used for each single mask taken in this study are shown, respectively.

SEQUENCES	MODEL	Accuracy	AUC
T2 nod	Random Forest	0.78	0.78
	Logistic Regression	0.78	0.78
	Support Vector Machine	0.78	0.17
DWI	Random Forest	0.86	0.89
	Logistic Regression	0.78	0.67
	Support Vector Machine	0.89	0.28
ADC	Random Forest	0.89	0.67
	Logistic Regression	0.67	0.67
	Support Vector Machine	0.78	0.67

**Table 4:** (**a**) Radiomics models (LR, RF, and SVM) and clinical nomograms absolute AUC and their comparison are reported. The analysis was performed using features obtained using only T2 sequences. The significant value was set at *p* ≤ 0.05. (**b**) Radiomics models (LR and RF) and clinical nomograms absolute AUC and their comparison are reported. The analysis was performed using features obtained using only DWI sequences. The significant value was set at *p* ≤ 0.05. (**c**) Radiomics models (LR and RF) and clinical nomograms absolute AUC and their comparison are reported. The analysis was performed using features obtained using only ADC sequences. The significant value was set at *p* ≤ 0.05.

**(a)**				
**Model Comparison**	**AUC Radiomics Model**	**AUC Nomogram**	**Z-Score**	***p*-Value**
LR vs. Briganti	0.89	0.79	0.833	0.405
LR vs. Partin	0.89	0.78	1.117	0.264
LR vs. MSKCC	0.89	0.78	1.132	0.258
LR vs. YALE	0.89	0.78	1.123	0.262
RF vs. Briganti	0.78	0.79	−0.345	0.730
RF vs. Partin	0.78	0.78	0	1
RF vs. MSKCC	0.78	0.78	0	1
RF vs. YALE	0.78	0.78	0	1
SVM vs. Briganti	0.17	0.79	1248.94	<0.05
SVM vs. Partin	0.17	0.78	1221.28	<0.05
SVM vs. MSKCC	0.17	0.78	1221.64	<0.05
SVM vs. YALE	0.17	0.78	1221.46	<0.05
**(b)**				
**Model Comparison**	**AUC radiomics model**	**AUC nomogram**	**Z-Score**	***p*** **-Value**
RF vs. Briganti	0.89	0.79	2.00	0.0455
RF vs. Partin	0.89	0.78	2.20	0.0278
RF vs. MSKCC	0.89	0.78	2.20	0.0278
RF vs. YALE	0.89	0.78	2.20	0.0278
LR vs. Briganti	0.671	0.79	1.733	0.083
LR vs. Partin	0.671	0.78	1.546	0.122
LR vs. MSKCC	0.671	0.78	1.529	0.126
LR vs. YALE	0.671	0.78	1.507	0.132
**(c)**				
**Model Comparison**	**AUC radiomics model**	**AUC nomogram**	**Z-score**	***p*-value **
LR vs. Briganti	0.67	0.79	−0.439	0.661
LR vs. Partin	0.67	0.78	0.039	0.969
LR vs. MSKCC	0.67	0.78	−0.028	0.978
LR vs. YALE	0.67	0.78	0.065	0.948
RF vs. Briganti	0.67	0.80	−1.27	0.205
RF vs. Partin	0.67	0.78	−1.11	0.268
RF vs. MSKCC	0.67	0.78	−1.06	0.290
RF vs. YALE	0.67	0.78	−1.05	0.295

## Data Availability

All data are available by request.
